# LAPAROSCOPIC UNCINATECTOMY: A MORE CONSERVATIVE APPROACH TO THE UNCINATE PROCESS OF THE PANCREAS

**DOI:** 10.1590/0102-6720201700020015

**Published:** 2017

**Authors:** Rodrigo Cañada SURJAN, Tiago BASSERES, Fabio Ferrari MAKDISSI, Marcel Autran Cesar MACHADO, José Celso ARDENGH

**Affiliations:** 1 Department of Surgery, School of Medicine, University of São Paulo, São Paulo, SP, Brazil

**Keywords:** Pancreas, Minimally invasive surgery, Laparoscopy, Neuroendocrine tumor.

## Abstract

**Background:**

The isolate resection of the uncinate process of the pancreas is a rarely described procedure but is an adequate surgery to treat benign and low grade malignancies of the uncinate process of the pancreas.

**Aim:**

To detail laparoscopic uncinatectomy technique and present the initial results.

**Method:**

Patient is placed in supine position with the surgeon between legs. Three 5-mm, one 10-mm and one 12-mm trocars were used to perform the isolated resection of the uncinate process of the pancreas. Parenchymal transection is performed with harmonic scalpel. A hemostatic absorbable tissue is deployed over the area previously occupied by the uncinate process. A Waterman drain is placed.

**Result:**

This procedure was applied to an asymptomatic 62-year-old male with biopsy proven low grade neuroendocrine tumor of the pancreatic uncinate process. A laparoscopic pancreaticoduodenectomy was proposed. During the initial surgical evaluation, intraoperative sonography was performed and disclosed that the lesion was a few millimeters away from the Wirsung. The option was to perform a laparoscopic uncinatectomy. Postoperative period until full recovery was swift and uneventful.

**Conclusion:**

Laparoscopic uncinatectomy is a safe and efficient procedure when performed by surgical teams with large experience in minimally invasive biliopancreatic procedures.

## RESUMO

**Racional:**

A ressecção isolada do processo uncinado do pâncreas é procedimento raramente relatado na literatura, mas consiste em operação adequada para o tratamento de tumores benignos e malignidades não-invasivas de baixo grau do processo uncinado do pâncreas.

**Objetivo:**

Detalhar a técnica da uncinectomia laparoscópica e apresentar os resultados iniciais.

**Método:**

Paciente é colocado em posição supina com o cirurgião entre as pernas. São utilizados três trocárteres de 5 mm, um de 10 mm e um de 12 mm para realizar a ressecção isolada do processo uncinado. A transecção parenquimatosa é realizada com bisturi harmônico. Tecido absorvível hemostático é implantado sobre a área anteriormente ocupada pelo processo uncinado. Dreno de Waterman é colocado sobre a área de superfície cruenta pancreática.

**Resultado:**

Este procedimento foi utilizado em um caso de tumor neuroendócrino de processo uncinado de pâncreas de baixo grau comprovado por biópsia guiada por ultrassonografia endoscópica. Ultrassonografia intra-operatória evidenciou lesão distando alguns milímetros do ducto de Wirsung, e assim optou-se pela realização de uncinectomia laparoscópica. A evolução pós-operatória até recuperação completa foi rápida e sem intercorrências.

**Conclusão:**

A uncinectomia por via laparoscópica é procedimento eficaz e seguro em equipes com experiência em cirurgia biliopancreática minimamente invasiva.

## INTRODUCTION

Surgical resection is the treatment of choice of pancreatic malignancies. Tumors of the head and uncinated process of the pancreas are usually resected by pancreaticoduodenectomy^1^ ^,^ ^2^ ^,^ ^7^. Since 1996, new techniques were designed in order to treat benign or low grade non-invasive pancreatic malignancies located in those anatomic locations^3^ ^,^ ^6^ ^,^ ^11^. The main objective was to preserve the duodenum and the common bile duct and thus reduce the morbidity of more extensive pancreatic resection. For tumors located specifically on the uncinate process, its isolated resection was developed^3^ ^,^ ^9^ ^,^ ^12^ ^,^ ^13^. This procedure has the additional advantages of preserving the Wirsung duct and the normal pancreatic juice flow to the duodenum and maximal pancreatic parenchymal preservation^13^.

Despite it´s clear advantages when compared to the pancreaticoduodenectomy, the pancreatic uncinatectomy has been rarely reported on the literature. The main reasons why this type of resection is not commonly used are the technical difficulties involved on performing this procedure, the risk of postoperative pancreatic leak and lesion to the main pancreatic duct while transecting the parenchyma^8^ ^,^ ^13^. 

If uncinatectomies were barely reported, it took more than 10 years to the first report of a laparoscopic resection of the uncinated process of the pancreas^5^. Since then, only two more articles described a complete anatomical isolated resection of the uncinated process of the pancreas^4^ ^,^ ^10^.

The objective of this study was to detail the laparoscopic uncinatectomy technique and present the initial results.

## METHOD

### Tecnique

**FIGURE 1:**
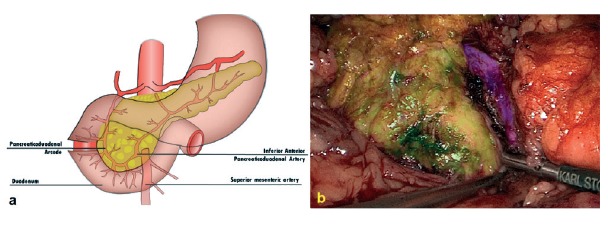
Technical details: A) pancreaticoduodenal arcade; B) color brushed areas: uncinate process in green, pancreatic head in yellow and superior mesenteric vein in purple.

Patient is placed in supine position with the surgeon between patient´s legs. Three 5-mm, one 10-mm and one 12-mm trocars are used to perform the isolated resection of the uncinate process of the pancreas. It is dissected away from the superior mesenteric vein and the duodenum, while preserving the inferior pancreaticoduodenal artery and the anterior pancreaticoduodenal arcade (Figure 1).

Parenchymal transection is performed with harmonic scalpel, a safe and effective method for cutting the pancreas^14^. Surgical specimen is retrieved inside a plastic retrieval bag through the 12-mm trocar incision that is enlarged to this purpose. A hemostatic absorbable tissue is deployed over the area previously occupied by the uncinate process. A Waterman drain is placed over the pancreatic raw surface area and brought out by the enlarged 12-mm trocar incision.

## RESULTS

**FIGURE 2:**
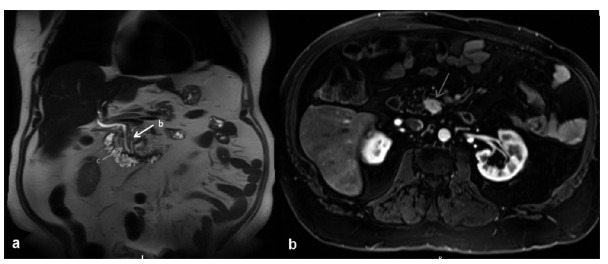
Magnetic resonance findings: A) coronal image showing tumor (red arrow); main pancreatic duct (yellow arrow); common bile duct (green arrow); B) axial image showing the tumor (red arrow)

This method was applied to treat a neuroendocrine tumor of the uncinate process of the pancreas on a patient in whom the initial idea was to perform a laparoscopic pancreaticoduodenectomy, but intraoperative sonography demonstrated that a parenchymal-sparing surgery would be possible. The patient, 62, had a gastric bypass one year before and presented with a 2.4 cm lesion on the uncinate process of the pancreas with close relation to the Wirsung duct on magnetic resonance. This lesion had been previously biopsied with endoscopic ultrasound guidance and pathology disclosed a neuroendocrine tumor (Figure 2).

**FIGURE 3:**
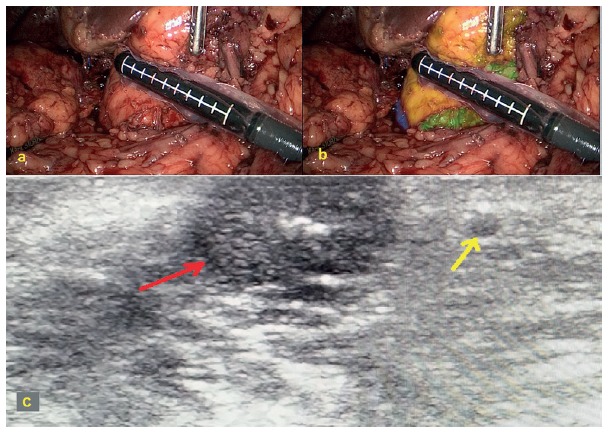
Intraoperative aspects: A) surgical field; B) color brushed areas: pancreatic head in yellow, uncinate process in green and duodenum in purple; c) intraoperative ultrasonography image disclosing the tumor (red arrow) and the main pancreatic duct (yellow arrow).

The initial propose was to perform a laparoscopic pancreaticoduodenectomy, but after taking down adherences from the previous procedure, performing a Kocher maneuver and exposing the head and uncinate process of the pancreas, an intraoperative sonography was performed and demonstrated that the tumor was a few millimeters away from the main pancreatic duct (Figure 3).

Operative time was 220 min. Estimated blood loss was 80 ml. Patient spent one day in the intensive care unit due to cardiological comorbidities. He was discharged from hospital on the 4^th^ postoperative day. Serum amylase levels remained in normal range after the procedure. He developed a grade A pancreatic fistula, and the drain was removed two weeks after surgery. Final pathology came out as a 2.1 cm pancreatic neuroendocrine tumor with free surgical margins. Eight months after the procedure the patient was asymptomatic with normal oral intake, bowel habits and glucose tolerance test.

## DISCUSSION

Tumors of the head and uncinated process of the pancreas are usually treated by pancreaticoduodenectomy. However, this is a major surgical procedure that requires resection of the duodenum, distal common bile duct and transection of the body of the pancreas, leading to the necessity of performing a biliodigestive derivation and a pancreaticojejunal (or pancreatogastric) anastomosis to digestive tract reconstruction. 

In the last two decades, different techniques have been developed with the intent to preserve the duodenum, common bile duct and upper part of the head of the pancreas while treating benign or noninvasive low grade malignancies of the head and uncinated process of the pancreas^6^ ^,^ ^13^. The advantages of these less extended procedures are preservation of normal pancreatic parenchyma, maintenance of physiological pancreatic juice and bile flow and lower postoperative morbidity^6^ ^,^ ^12^ ^,^ ^13^.

The isolate resection of the uncinated process of the pancreas, or so called uncinatectomy, is an adequate option to treat noninvasive tumors located on the uncinated process of the pancreas. Despite being a less extensive procedure than a pancreaticoduodenectomy, this is a challenging surgery due to the close relation of the uncinated process to important vascular pedicles (such as the inferior mesenteric vein). As a result, it was not until recently that this procedure started to be reported in the literature and only in few reports^3^ ^,^ ^8^ ^,^ ^9^ ^,^ ^12^ ^,^ ^13^.

The first laparoscopic resection of the uncinated process of the pancreas would not be reported before 2009^5^. Since then, this procedure has rarely been reported in the literature^4^ ^,^ ^10^ ^,^ ^11^. Nevertheless, the laparoscopic uncinatectomy is an effective and less invasive alternative than a pancreaticoduodenectomy to treat neoplasms of the uncinated process of the pancreas when oncologically appropriate.

Here is described a patient that initially would be submitted to a laparoscopic pancreaticoduodenectomy to treat a neuroendocrine tumor of the uncinated process of the pancreas that on preoperative image assessment was close to the main pancreatic duct. However, during intraoperative sonographic evaluation, it was noticed that the tumor was a few millimeters away from the main pancreatic duct, leaving the opportunity to preserve the head of the pancreas. The option was to perform a laparoscopic resection of the uncinated process of the pancreas, with the advantages of maximal preservation of pancreatic parenchyma (with its endocrine and exocrine functions), lower postoperative morbidity and preservation of the biliopancreatic and duodenal anatomy^6^ ^,^ ^12^ ^,^ ^13^. The awareness of this possibility and an experienced and prepared surgical team permitted to change the initial intended procedure to a less extended surgery, with swift and uneventful postoperative recovery.

## CONCLUSION

 Pancreatic parenchymal sparing procedures are oncologically effective procedures for nonmalignant and low grade malignancies of the pancreas that should be cogitated in order to avoid postoperative exocrine and endocrine pancreatic insufficiency and unnecessary extended procedures. The laparoscopic uncinatectomy is a feasible and safe surgery that must be considered in this context.
